# Prehospital factors of survival to hospital admission in blunt traumatic out-of-hospital cardiac arrest: a nationwide 11-year study

**DOI:** 10.1016/j.resplu.2025.101086

**Published:** 2025-09-04

**Authors:** Thanakorn Laksanamapune, Welawat Tienpratarn, Chaiyaporn Yuksen, Danaiporn Suktarom, Phunyapat Pankeaw, Irada Somawong, Sittichok Leela-Amornsin

**Affiliations:** a Department of Emergency Medicine, Faculty of Medicine, Ramathibodi Hospital, Mahidol University, Bangkok 10400, Thailand; b Chakri Naruebodindra Medical Institute, Faculty of Medicine, Ramathibodi Hospital, Mahidol University, Samut Prakan, Thailand

**Keywords:** Traumatic cardiac arrest, Blunt trauma, Out-of-hospital cardiac arrest (OHCA), Emergency medical services (EMS), Prehospital care, Prognostic factors, Survival

## Abstract

**Background:**

Blunt traumatic out-of-hospital cardiac arrest (TOHCA) is consistently associated with poor survival outcomes. Although the prehospital interventions may influence prognosis. This study aimed to identify independent prehospital factors associated with survival to hospital admission among patients of blunt TOHCA in Thailand.

**Methods:**

This retrospective cohort study used nationwide data from the Information Technology of Emergency Medical System database between 2012 and 2022. Patients of all ages with blunt TOHCA who received prehospital resuscitation and were transported to the emergency department were included. The primary outcome was survival to hospital admission. Multivariable logistic regression was used to identify associated prehospital factors.

**Results:**

Of 18,612 patients with blunt TOHCA, 3,004 (16.1 %) survived to hospital admission. The survival rate declined from 28 % in 2012 to 13 % in 2022. Several independent factors associated with improved survival, including external bleeding control (adjusted odds ratio [aOR] 1.20, 95 % confidence interval [CI]: 1.03–1.40), endotracheal intubation (aOR 2.12, 95 % CI: 1.74–2.58), intravenous fluid administration (aOR 1.65, 95 % CI: 1.32–2.06), defibrillation (aOR 2.40, 95 % CI: 1.99–2.90), longer on-scene time (aOR 1.03, 95 % CI: 1.02–1.03), and head/neck injuries (aOR 1.30, 95 % CI: 1.11–1.51). In contrast, longer hospital-to-scene distances, chest/clavicle injuries, and open/closed fractures were associated with decreased odds of survival.

**Conclusion:**

This study highlights critical challenges in the prehospital management of blunt TOHCA in Thailand. Timely and appropriate interventions, including bleeding control, airway management, intravenous fluid administration, and defibrillation, may enhance survival outcomes.

## Background

Traumatic out-of-hospital cardiac arrest (TOHCA) represents a life-threatening condition among injured patients with dismal survival outcomes.^1^, ^2^, ^3^, ^4^ The majority of TOHCA cases are attributed to blunt trauma,^1^, ^2^, ^3^, ^4^, ^5^ which consistently demonstrates lower survival rates when compared to penetrating trauma.^2^, ^4^, ^6^ Penetrating and blunt trauma differ significantly in pathophysiology and outcomes, with blunt trauma often causing multisystem injuries and poorer survival (2.3 %), while penetrating trauma, despite higher rates of traumatic cardiac arrest, has better survival (10.6 %) due to treatable vascular injuries.^7^ Blunt trauma, often resulting from road traffic injuries or falls from height, is a leading cause of TOHCA and is associated with severe complications such as major hemorrhage, tension pneumothorax, and severe traumatic brain injury. These complications create a complex physiological challenge that often results in poor survival despite advances in prehospital care systems.^1^, ^2^ TOHCA from blunt trauma necessitates immediate, specialized interventions at the scene, as conditions like hypoxia, hypovolemia, and airway obstruction are common contributors to mortality. Emergency medical services (EMS) systems, particularly those equipped with advanced life support (ALS) capabilities, play a vital role in initiating life-saving measures and ensuring rapid transport to definitive care.^2^, ^4^, ^5^

A Previous study highlights the poor prognosis of blunt TOHCA, with survival rates as low as 3 %,^8^ even under standardized strategies and rapid EMS response. Prognostic indicators with poor survival outcomes included pulseless electrical activity or asystole as the initial rhythm, unwitnessed arrests, prolonged time to hospital arrival, and severe multisystem injuries, often caused by road traffic injuries or falls. The witnessed arrests and shorter transport times are linked to better outcomes, while aggressive interventions like thoracotomy and epinephrine administration show limited success and require selective application.^8^

Despite advancements, a significant gap persists in understanding prognostic factors, the role of prehospital procedures, particularly within Thailand's EMS system, and their influence on blunt TOHCA survival. To address this, we conducted a nationwide retrospective cohort study aimed to identify prognostic factors associated with survival to hospital admission in patients of all age groups experiencing blunt TOHCA.

## Methods

### Study design and setting

This retrospective observational cohort study, aimed to identify prognostic factors for survival to hospital admission in blunt TOHCA patients. In the setting of Thailand, EMS begins when the dispatcher detects the level of an emergency by telephone triage. Second, the dispatch center should assign the EMS team to respond, provide prehospital emergency care, and transport the patient to an appropriate healthcare facility. All data, including the emergency chief complaint, dispatch instructions, patient assessments, and prehospital interventions, were recorded and collected in a unified national database from the Information Technology of Emergency Medicine System (ITEMS). This study utilizes the data for analysis from an 11-year ITEMS program of the National Institute of Emergency Medicine in Thailand.

The prehospital emergency care of TOHCA is provided through a tiered response system involving both basic and advanced teams. Typically, basic teams arrive at the scene first and initiate chest compressions, provide basic airway support, and apply automated external defibrillators (AED) when indicated. These teams are subsequently supported by Advanced Life Support (ALS) units, which consist of paramedics, emergency nurses, and emergency physicians. All members are trained and authorized to perform standardized advanced procedures, including endotracheal intubation, intravenous fluid administration, defibrillation, and the administration of emergency medications.

National EMS protocols, adapted from Prehospital Trauma Life Support guidelines, emphasize a “scoop and run” approach, with a recommended on-scene time of less than 10 minu.^9^ Under the current national policy, termination of resuscitation is not permitted in the field; therefore, all TOHCA patients must be transported to the nearest emergency department (ED) regardless of return of spontaneous circulation status.

This study was ethically approved by the Faculty of Medicine, Committee on Human Rights Related to Research Involving Human Subjects, Ramathibodi Hospital, Mahidol University (COA. NO MURA2025/4). Given the study’s retrospective nature, the requirement for obtaining informed consent from individual patients was waived.

### Participants

Blunt TOHCA patients who received prehospital resuscitation and were transported to the ED between January 2012 and December 2022 were included. The exclusion criteria were TOHCA patients with missing ED outcome data, defined as missing information on survival to hospital admission.

### Variables and outcome measurement

The 11-year prehospital data were collected from the ITEMS database for analysis, including demographic parameters (age, sex), EMS operational time intervals parameters (response time, on-scene time, and transport time), and distances (hospital-to-scene, scene-to-hospital) were recorded for calculation. In addition, on-scene physiologic parameters were documented, along with types of fractures (closed fracture, open fracture, dislocation) and exsanguination bleeding (external bleeding). The location of injuries was categorized by body regions (head/neck, face, spine, chest/clavicle, abdomen, pelvis, extremity, multiple injuries). Moreover, data on prehospital interventions and the level of care provided were collected. This included bleeding control, endotracheal intubation, intravenous fluid administration, use of an automated external defibrillator (AED) or manual defibrillation, administration of adrenaline, and involvement of emergency medical services (EMS) teams with advanced life support (ALS) capabilities.

The primary outcome was survival to hospital admission, defined as patients who were subsequently admitted to an inpatient ward or intensive care unit after emergency treatment in the ED. Patients who died in the ED without hospital admission were classified as non-survivors.

### Definitions

**Blunt traumatic out-of-hospital cardiac arrest** is cardiac arrest caused by traumatic events at the scene or prehospital phase, including traffic crash,^10^ fall, collision,^11^ and blunt mechanism in any location of the bodies.

**Response time** is the duration between an EMS team receiving a call from a dispatcher to arrival an incident scene.^12^

**On-scene time** is the time duration the EMS team spends at the incident scene before transporting the patient to a hospital or appropriate facility.^13^

**Transport time** is the total time from the incident scene to the hospital or appropriate facility.^13^

### Statistical analysis

The statistical analyses were performed by STATA version 16.1. All prognostic factors were analyzed to demonstrate the baseline characteristics of patients, categorized into two groups of results: those who survived to hospital admission and those who did not survive. Furthermore, all factors were subjected to multivariable logistic regression analysis to evaluate their potential association with survival outcomes.

Categorical variables were analyzed using chi-squared tests and presented as frequency and percentage. Continuous variables with non-normal distribution were compared using the Mann-Whitney *U* test and reported as median with interquartile range (IQR).

Multivariable logistic regression analysis was conducted to identify independent prognostic factors associated with survival to hospital admission in TOHCA. EMS operational time intervals were included as continuous variables, while their dichotomized forms were excluded to ensure model integrity and reduce redundancy. Multicollinearity was assessed using the Variance Inflation Factor (VIF), with all variables demonstrating acceptable values, indicating no significant collinearity. Results are presented as crude odds ratios (cOR) and adjusted odds ratios (aOR), with corresponding 95 % confidence intervals (CIs) and p-values. A p-value of less than 0.05 was considered statistically significant.

Patients with missing ED outcome data were excluded from the analysis. No imputation was applied, as the remaining sample size was sufficiently large to ensure statistical robustness.

This manuscript was prepared in accordance with the STROBE (Strengthening the Reporting of Observational Studies in Epidemiology) statement of cohort studies.^14^

## Results

### The study population (participants)

**Fig. 1:**
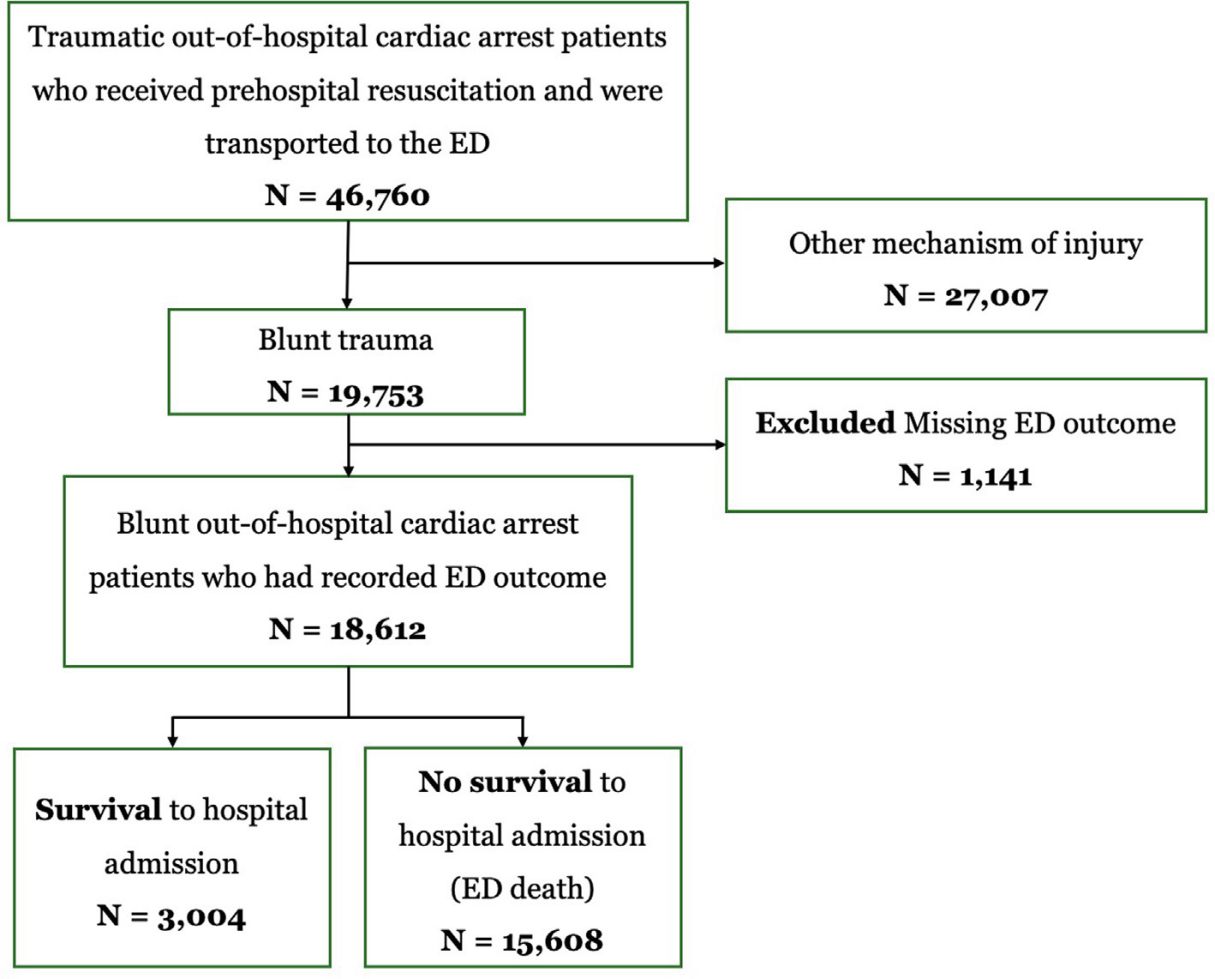
Study flow of patients’ inclusion. ED: Emergency department.

This study retrieved and analyzed data from January 2012 to December 2022. Encompassing an 11-year period, a total of 46,760 TOHCA patients were identified. After excluding 27,007 patients with non-blunt trauma and 1,141 patients (5.78 %) with missing ED outcomes. Of the 18,612 patients with blunt TOHCA included in the analysis, 3,004 (16.14 %) survived to hospital admission, while 15,608 (83.86 %) died at the ED. (Fig. 1).

### The trend of survival to hospital admission in blunt TOHCA in Thailand over 11 years

**Fig. 2:**
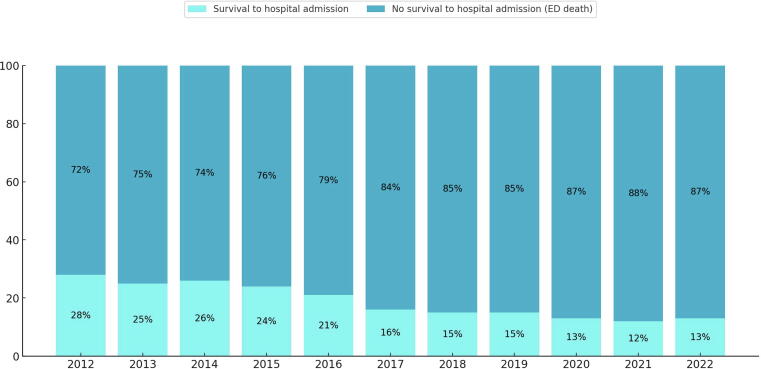
The trend of survival to hospital admission of blunt traumatic out-of-hospital cardiac arrest (TOHCA) patients in Thailand from 2012 to 2022 (11 years). ED: Emergency department.

The trend of the proportion of survival to hospital admission versus no survival to hospital admission (ED death) among blunt TOHCA patients in Thailand from 2012 to 2022. Over this 11-year period, there is a noticeable and consistent increase in the percentage of ED death, including contextualizing with regional factors, system-level operations, and the specific nature of trauma in the setting of Thailand, accompanied by a corresponding decrease in the percentage of survival. Specifically, survival decreased from 28 % in 2012 to 13 % in 2022, representing a reduction of 15 percentage points. This shift indicates a declining trend in survival rates. The most significant drops in survival appear between 2016 and 2018, as well as between 2019 and 2021 (Fig. 2).

### Baseline characteristics of blunt TOHCA patients in Thailand (descriptive and outcome data)

A total of 18,612 patients with blunt TOHCA were included. The majority were male (79.5 %), with a median age of 37 years; children (≤15 years) accounted for 6.9 % of cases. The median of response, on-scene, and transport times were 9, 5, and 7 min, respectively. The median distance from hospital to scene and scene to hospital was 6 km.

Regarding injury characteristics, closed fractures (36.6 %) and dislocations (32.4 %) were common, while open fractures occurred in 2.7 %. Internal bleeding (72.3 %) was more frequent than external bleeding (27.8 %). The most frequent injury site was the head and neck (77.7 %), followed by the chest/clavicle (7.0 %), abdomen (6.0 %), and pelvis (3.0 %).

Prehospital interventions were widely performed: endotracheal intubation (96.1 %), automated external defibrillator/defibrillation use (96.0 %), intravenous fluid administration (94.0 %), and external bleeding control (68.5 %). Adrenaline was administered in 69.2 % of cases. Almost all patients were managed by advanced life support (ALS) EMS teams (99.9 %) (Table 1).

**Table 1:** Baseline characteristics of blunt traumatic out-of-hospital cardiac arrest (TOHCA) patients.

**Prognostic factors**	**N (%)**
**Sex**	
Male	14,401 (79.45)
**Age (years)**	
Median (IQR)	37 (24, 53)
Children (≤15 years)	1,268 (6.87)
Adult (>15 years)	17,188 (93.13)
**EMS operational time intervals (minutes):** Median (IQR)	
Response time	9 (6, 13)
On-scene time	5 (3, 8)
Transport time	7 (4, 10)
**Distance (km):** Median (IQR)	
Hospital-to-scene	6 (3, 10)
Scene-to-hospital	6 (3, 10)
**Type of Fracture**	
Closed fracture	6,084 (36.59)
Open fracture	446 (2.68)
Dislocation	5,382 (32.37)
**Exsangunation bleeding**	
External bleeding	4,669 (27.75)
Internal bleeding	12,159 (72.25)
**Location of injury**	
Head/neck	13,631 (77.74)
Spine	272 (1.55)
Chest/clavicle	1,224 (6.98)
Abdomen	1,050 (5.99)
Pelvis	528 (3.01)
Multiple injury	190 (1.08)
**Prehospital interventions**	
External bleeding control	3,196 (68.45)
Endotracheal intubation	17,696 (96.09)
Intravenous fluid administration	17,139 (94.04)
AED/Defibrillation	17,875 (96.04)
Adrenaline use	12,887 (69.24)
**EMS Level Operation**	
ALS	18,589 (99.88)
**ED outcome**	
Survival to hospital admission	3,004 (16.14)
No survival to hospital admission (ED death)	15,608 (83.86)

EMS: Emergency medical services, km: kilometer, AED: Automated external defibrillator, ALS: Advanced life support, ED: Emergency department, IQR: interquatile range.

### Multivariable analysis for independent prognostic factors associated with survival to hospital admission in blunt TOHCA (main results)

**Fig. 3:**
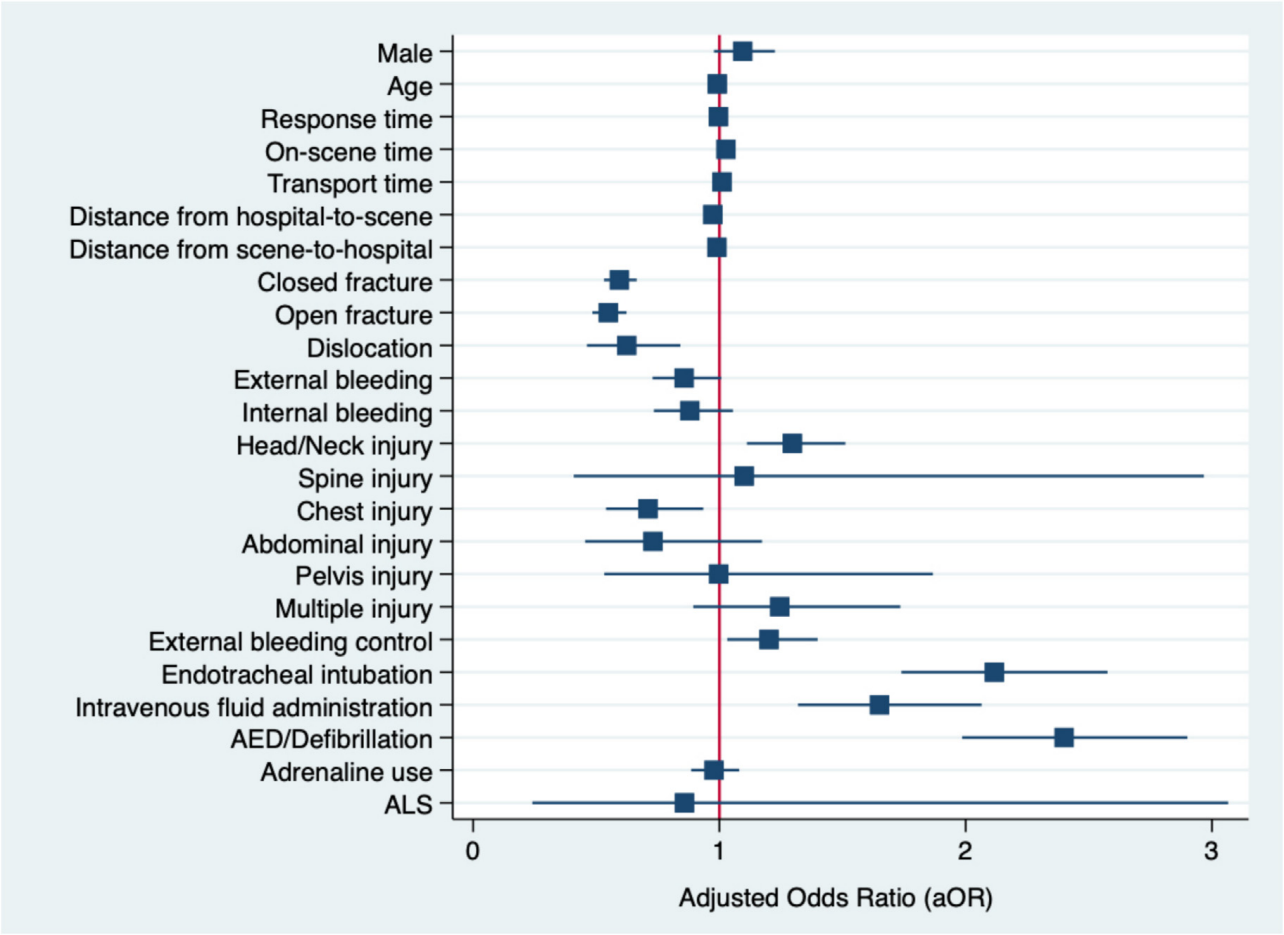
Coefficient plot graph of independent prognostic factors of survival to hospital admission in blunt traumatic out-of-hospital cardiac arrest (TOHCA) patients. AED: Automated external defibrillator, ALS: Advanced life support.

The multivariable logistic regression analysis revealed several factors that were associated with the likelihood of survival to hospital admission in blunt TOHCA patients. Some factors were associated with better outcomes, while others reduced the chances of survival (Table 2, Fig. 3).

**Table 2:** Multivariable logistic regression analysis of independent prognostic factors of survival to hospital admission in blunt traumatic out-of-hospital cardiac arrest (TOHCA) patients.

**Prognostic factors**	**cOR (95 % CI)**	**aOR (95 %CI)**
**Sex**		
Male	1.02 (0.93–1.13)	1.09 (0.98–1.23)
**Age (year)**	0.99 (0.99–1.00)	0.99 (0.99–0.99)
**EMS operational time intervals (minutes)**		
Response time	0.98 (0.98–0.99)	1.00 (0.99–1.01)
On-scene time	1.02 (1.02–1.03)	1.03 (1.02–1.03)
Transport time	0.99 (0.99–1.00)	1.01 (1.00–1.02)
**Distance (km)**		
Hospital-to-scene	0.97 (0.97–0.98)	0.97 (0.96–0.99)
Scene-to-hospital	0.98 (0.97–0.99)	0.99 (0.97–1.01)
**Type of fracture**		
Closed fracture	0.76 (0.70–0.84)	0.59 (0.53–0.66)
Open fracture	0.72 (0.66–0.80)	0.55 (0.49–0.62)
Dislocation	0.98 (0.75–1.27)	0.62 (0.46–0.84)
**Exsanguination bleeding**		
External bleeding	0.79 (0.72–0.86)	0.86 (0.73–1.01)
Internal bleeding	0.83 (0.74–0.94)	0.88 (0.74–1.05)
**Location of injury**		
Head/neck	1.24 (1.12–1.37)	1.30 (1.11–1.51)
Spine	1.18 (0.52–2.68)	1.10 (0.41–2.97)
Chest/clavicle	0.65 (0.54–0.79)	0.71 (0.54–0.94)
Abdomen	0.72 (0.50–1.04)	0.73 (0.45–1.17)
Pelvis	0.84 (0.51–1.38)	1.00 (0.53–1.87)
Multiple injury	0.88 (0.68–1.14)	1.25 (0.89–1.73)
**Prehospital interventions**		
External bleeding control	1.31 (1.20–1.42)	1.20 (1.03–1.40)
Endotracheal intubation	2.06 (1.74–2.44)	2.12 (1.74–2.58)
Intravenous fluid administration	1.39 (1.15–1.67)	1.65 (1.32–2.06)
AED/Defibrillation	2.37 (2.02–2.79)	2.40 (1.99–2.90)
Adrenaline use	0.95 (0.87–1.03)	0.98 (0.89–1.08)
**EMS Level Operation**		
ALS	0.91 (0.31–2.69)	0.86 (0.24–3.07)

EMS: Emergency medical services, km: kilometer, AED: Automated external defibrillator, ALS: Advanced life support, cOR: crude odds ratio, aOR: adjusted odds ratio.

Independent prognostic factors significantly associated with increased odds of survival to hospital admission included proximity to the hospital and the provision of specific prehospital interventions. Patients located closer to the hospital had better survival outcomes, that associated with higher odds of survival (aOR 0.97, 95 % CI: 0.96–0.99, *p* = 0.001). Additionally, longer on-scene time was positively associated with survival (aOR 1.03, 95 % CI: 1.02–1.03, *p* < 0.001).

Key prehospital interventions were also associated with improved survival. These included external bleeding control (aOR 1.20, 95 % CI: 1.03–1.40, *p* = 0.018, endotracheal intubation (aOR 2.12, 95 % CI: 1.74–2.58, *p* < 0.001), and AED/defibrillation (aOR 2.40, 95 % CI: 1.99–2.90, *p* < 0.001). Head and neck injuries were associated with higher odds of survival (aOR 1.30, 95 % CI: 1.11–1.51, *p* = 0.001).

Conversely, specific injury patterns were linked to poorer outcomes. Patients with chest or clavicle injuries (aOR 0.71, 95 % CI: 0.54–0.94, p-0.015), open fractures (aOR 0.55, 95 % CI: 0.49–0.62, *p* < 0.001), or closed fractures (aOR 0.59, 95 % CI: 0.53–0.66, *p* < 0.001) had significantly reduced odds of survival to hospital admission.

## Discussion

This study provides a comprehensive analysis of blunt TOHCA patients over an 11-year period in Thailand, focusing on baseline characteristics and outcomes following prehospital resuscitation interventions by EMS teams. These results highlight the importance of timely and targeted prehospital interventions in improving outcomes. Key procedures include external bleeding control, endotracheal intubation, intravenous fluid administration, defibrillation, and adequate on-scene time. These findings suggest that prehospital interventions should be initiated at the scene before transporting the patient to the ED.

In a previous multi-center study in Thailand using the Pan-Asian Resuscitation Outcomes Study database, the 30-day survival rate for non-traumatic arrest was approximately 3.4 %, based on data from three centers in Bangkok and Chiang Mai.^15^ In contrast, our study focused on blunt TOHCA, which showed a lower survival rate to hospital admission and a declining trend over time. Our survival rate is similar to the previous studies in blunt TOHCA. Martin et al.^16^ showed that 23 % of patients who survived to ED in pulseless electrical activity rhythm, and Moriwaki et al.^8^ showed 18 % of patients who survived to hospital admission in patients with and without ED thoracotomy.

Previous studies on adult TOHCA resulting from blunt, penetrating, or burn injuries^16^ and our primary research encompassing all mechanisms of injury in adult and pediatric TOHCA^5^ emphasize the critical role of prehospital interventions and rapid transportation in improving survival outcomes. EMS teams play a pivotal role in providing these life-saving interventions^5^, ^6^, ^8^, ^16^, ^17^. Similarly, our subgroup study focused on blunt trauma demonstrated that survivors were more likely to receive prehospital interventions,^18^ including controlling external bleeding, inserting an endotracheal tube, administering intravenous fluid, and applying AED/defibrillation, with shorter hospital-to-scene distances and appropriate on-scene time, although these apparent benefits may be subject to confounding by indication. It seems that hypoxia, often caused by apnea from traumatic brain injury, was the most common suspected reason for TOHCA among survivors, followed by hypovolemia from major hemorrhage,^6^ for which the prehospital interventions may benefit. Nonetheless, some studies, including trauma registries in the United States^4^ and a systematic review and *meta*-analysis on prehospital TOHCA by Vianen et al.,^19^ have reported that prehospital interventions were not significantly associated with improved survival. These findings suggest that survival may depend more on performing only essential prehospital procedures and optimizing the on-scene time to ensure rapid transport to definitive care in trauma centers.

TOHCA patients with an initial shockable rhythm, as reported consistently in previous studies, have significantly higher survival rates.^16^, ^19^, ^20^, ^21^, ^22^ Although initial rhythms were not recorded in the ITEMS database, we used defibrillation/AED use as a surrogate marker. This likely indicates shockable rhythms such as ventricular fibrillation or pulseless ventricular tachycardia. While this proxy may reflect arrest aetiology more than treatment effect, we acknowledge its limitations in our interpretation. In addition, our study found no significant association between adrenaline administration and survival in TOHCA patients. While earlier studies suggested limited benefit of adrenaline in TOHCA,^19^, ^20^ a post-hoc analysis of the PARAMEDIC2 trial reported improved survival to hospital admission with adrenaline. However, this did not extend to long-term survival or neurological outcomes.^23^ These contrasting findings highlight the role of adrenaline in TOHCA and the need for further investigation. Although point-of-care ultrasound, including the extended focused assessment with sonography for trauma, is increasingly used to detect reversible causes in trauma, its role in TOHCA remains uncertain. Current evidence, including ILCOR 2020^24^ and a recent systematic review,^25^ supports its potential utility but highlights very low certainty and high risk of bias. These suggest that ultrasound should not interrupt chest compressions or delay critical interventions. Furthermore, in Thailand, the administration of blood at the scene of TOHCA is not currently practiced, reflecting a significant gap compared to EMS systems in other countries where prehospital blood transfusion^6^, ^16^, ^19^ has been implemented to address hypovolemia, especially major hemorrhage in blunt TOHCA. However, previous studies have indicated that prehospital blood administration is not a significant prognostic factor for survival in TOHCA.^6^, ^16^ Although EFAST and prehospital blood transfusion were not variables included in our dataset, the observed survival benefit from other timely prehospital interventions supports the broader need to enhance prehospital trauma assessment and resuscitation strategies in Thailand.

Conversely, certain injury locations were significantly associated with poor survival. Patients with chest or clavicle injuries and those with open or closed fractures had lower odds of survival to hospital admission. These findings align with previous studies showing that blunt thoracic trauma, such as pneumothorax or hemothorax, is associated with high mortality.^8^, ^26^, ^27^ Clavicle fractures often indicate severe trauma and frequently accompany upper rib fractures,^28^ while chest injuries impair ventilation and resuscitation success.^27^ Similarly, long bone fractures suggest high-energy trauma with risk of major hemorrhage,^29^, ^30^, ^31^ underscoring the need for targeted prehospital strategies.^32^, ^33^

The strength of our study lies in its comprehensive analysis of blunt TOHCA over an 11-year period, addressing a critical yet underrepresented subset of traumatic cardiac arrest. By leveraging large, nationwide real-world data from prehospital settings in Thailand, the study highlights the impact of specific prehospital interventions. Its findings are directly applicable to EMS practices in resource-limited settings and provide valuable insights for improving trauma care systems.

This study has several limitations. First, as a retrospective analysis, it is susceptible to biases from incomplete or inaccurate data. Excluding patients with missing primary outcome data may introduce selection bias, particularly if the missingness was not random. Although the missing data accounted for only 5 %, its impact on the results cannot be entirely ruled out. Additionally, the overall proportion of missingness across variables ranged from 0 % to 10.7 % and was considered relatively low. Second, the ITEMS database lacked critical variables such as initial cardiac rhythm, bystander CPR, and detailed prehospital interventions (needle thoracostomy for pneumothorax decompression),^32^, ^33^ limiting the ability to assess their prognostic value. Third, due to the absence of regional population data and EMS catchment information, we could not calculate incidence rates per population. Additionally, survival to hospital discharge was also unavailable. Although a decline in survival over time was observed despite improvements in trauma care, the underlying causes remain unclear and likely multifactorial. These limitations underscore the need for prospective studies to understand the prognostic impact of specific prehospital interventions and refine trauma care strategies, particularly in low- and middle-income settings like Thailand. Collaborative efforts between emergency physicians, EMS researchers, and EMS providers are crucial for addressing gaps in care and improving survival outcomes in blunt TOHCA.

## Conclusion

This study highlights the critical challenges in prehospital management of blunt TOHCA in Thailand. Our findings indicate that timely and targeted prehospital interventions such as external bleeding control, airway management, intravenous fluid administration, and defibrillation were associated with increased odds of survival to hospital admission. Strengthening these interventions and EMS system readiness may improve patient outcomes.

## Declarations

### Ethical approval and consent to participate

Ethical approval for this study was obtained from the Human Research Ethics Committee, Faculty of Medicine Ramathibodi Hospital, Mahidol University, granted on January 2, 2025 (IRB COA. MURA2025/4). The Institutional Review Boards in Mahidol University are in full compliance with the International Guidelines for Human Research Protection including the Declaration of Helsinki, The Belmont Report, CIOMS Guidelines, and the International Conference on Harmonization in Good Clinical Practice (ICH-GCP). All personal identification was removed and replaced with study IDs. Given the study’s retrospective nature, the requirement for obtaining informed consent from individual patients was waived.

### Consent for publication

Not applicable.

### Availability of data and material

The datasets generated during and/or analysed during the current study are available from the corresponding author on reasonable request.

### Competing interests

The author(s) declare no competing interests.

### Authors’ contributions

TL, WT, and SL wrote the main manuscript. TL, WT and SL initiated the research question and completed the IRB submission. TL, DS, PP, and IS performed data collection. WT and SL wrote the introduction, results, and discussion sections. TL and WT wrote the methods section, performed data analysis, and made tables and figures. WT and SL reviewed the manuscript. WT addressed the corresponding author. CY is an associate professor and research advisor at Mahidol University who provided guidance to the manuscript. All authors reviewed the final manuscript.

## CRediT authorship contribution statement

**Thanakorn Laksanamapune:** Writing – original draft, Resources, Project administration, Methodology, Investigation, Formal analysis, Data curation, Conceptualization. **Welawat Tienpratarn:** Writing – review & editing, Writing – original draft, Supervision, Methodology, Formal analysis, Conceptualization. **Chaiyaporn Yuksen:** Supervision. **Danaiporn Suktarom:** Data curation. **Phunyapat Pankeaw:** Data curation. **Irada Somawong:** Data curation. **Sittichok Leela-Amornsin:** Writing – review & editing, Supervision, Conceptualization.

## Funding

No funding was obtained for this study.

## Declaration of competing interest

The authors declare that they have no known competing financial interests or personal relationships that could have appeared to influence the work reported in this paper.
